# The dual role of intestinal parasites in shaping human gut microbiota: distinguishing helminthic and protozoan dynamics

**DOI:** 10.1186/s13099-026-00815-7

**Published:** 2026-03-02

**Authors:** Siti Farah Norasyikeen Sidi Omar, Romano Ngui, Timothy Jinam, Sam Froze Jiee, Hee Siong Lim, Paul Cliff Simon Divis, Lesley Maurice Bilung, Yvonne Ai Lian Lim

**Affiliations:** 1 Department of Paraclinical Sciences, Faculty of Medicine and Health Sciences, Universiti Malaysia Sarawak, Kota Samarahan, Sarawak, 94300 Malaysia; 2 Department of Community Medicine and Public Health, Faculty of Medicine and Health Sciences, Universiti Malaysia Sarawak, Kota Samarahan, Sarawak, 94300 Malaysia; 3 Malaria Research Centre, Faculty of Medicine and Health Sciences, Universiti Malaysia Sarawak, Kota Samarahan, Sarawak, 94300 Malaysia; 4 Resource Biotechnology Programme, Faculty of Resource Science and Technology, Universiti Malaysia Sarawak, Kota Samarahan, Sarawak, 94300 Malaysia; 5 Department of Parasitology, Faculty of Medicine, Universiti Malaya, 50603 Kuala Lumpur, Malaysia

**Keywords:** Gut microbiota, Intestinal parasites, Helminths, Protozoa, Polyparasitism, soil-transmitted helminths, Dysbiosis, Parasitic infections, Human gut, Parasite-microbiota interactions

## Abstract

The human gut microbiota is essential for supporting the host’s health and immune system. Imbalances in this microbiota, known as dysbiosis, are associated with numerous inflammatory and autoimmune diseases. Despite extensive research on bacterial components, the significant modulatory role of eukaryotic gut inhabitants, particularly intestinal parasites, remains largely overlooked. While traditionally viewed as pathogens, emerging evidence highlights a profound duality in their role, suggesting they are integral and active modulators of the gut microbiota and challenging the conventional view of parasitism. For instance, helminths often induce microbial diversity and promote the expansion of anti-inflammatory microbes. Conversely, pathogenic protozoa are generally associated with reduced microbial diversity, fostering the growth of pathobionts and leading to significant gut dysbiosis. However, these interactions are highly context-dependent, and a comprehensive understanding is hindered by varied findings and limited data, particularly concerning protozoan infections. Furthermore, while mechanistic evidence is robust, human intervention studies remain limited. This narrative review synthesises the complex interrelationship between intestinal parasites and the human gut microbiota, distinguishing the distinct dynamics of helminthic and protozoan infections. Specifically, this review examines the inherent duality of intestinal parasites by investigating parasite-specific factors, including species, parasite burden, and coinfections, which can impact gut microbial composition and function. Ultimately, this review provides a comprehensive framework for understanding the profound influence of the gut ecosystem, shifting the paradigm beyond solely pathogenic views.

**Supplementary Information:**

The online version contains supplementary material available at 10.1186/s13099-026-00815-7.

## Introduction

Intestinal parasitic infections (IPIs), caused by helminths and protozoa, remain a major global health issue [1, 2]. Around 1.5 billion people worldwide are infected with helminths, while 500 million are affected by pathogenic protozoa [3, 4]. Among helminths, the soil-transmitted helminths (STH) *Ascaris lumbricoides*, *Trichuris trichiura*, and hookworms (*Necator americanus*, *Ancylostoma duodenale*) are the most prevalent [2]. Meanwhile, pathogenic intestinal protozoa such as *Giardia duodenalis*, *Entamoeba histolytica*, and *Cryptosporidium* spp. are likewise frequently implicated in human disease [2, 5].

Traditionally, parasites have been conceptualised solely within a pathogenic paradigm. However, emerging research increasingly demonstrates that intestinal parasites play an integral role in shaping the host gut microbiota, acting not only as disease-causing agents but also as modulators of microbial and immune homeostasis [6, 7]. Certain parasite exposure has been linked to protection against autoimmune and allergic conditions such as type 1 diabetes mellitus, multiple sclerosis, and inflammatory bowel disease (IBD) [8]. This evolving understanding is robustly substantiated by the “Old Friends Hypothesis,” which posits that exposure to diverse microorganisms, including parasites, is pivotal for immune maturation and regulation [9]. However, this hypothesis remains subject to debate, with ongoing controversy regarding dose-response effects, population-specific applicability, and other factors that underscore the intricate complexity of the host-parasite-microbiota interplay.

This dual role highlights the long-standing coevolutionary relationship between intestinal parasites and the human gut ecosystem. The gut microbiota itself represents a complex consortium of bacteria, archaea, viruses, and eukaryotes (e.g., fungi and protozoa) [10]. While bacterial components have been extensively characterised in relation to health and disease [11, 12], eukaryotic members, particularly intestinal parasites, are still primarily viewed through their detrimental impacts [13]. Their potential as modulators of immune responses and contributors to gut homeostasis is frequently overlooked [7].

This narrative review synthesises current evidence on the multifaceted interactions between intestinal parasites and the human gut microbiota, specifically elucidating distinct dynamics during helminthic and protozoan infections, highlighting species-specific effects, infection burden, and coinfections as key determinants of microbial outcomes. By exploring this inherent “duality”, encompassing both pathogenic consequences and potential modulatory roles, this review seeks to provide a comprehensive framework for understanding the influence of intestinal parasites on the human gut ecosystem.

## Methods

This narrative review synthesises current evidence on interactions between intestinal parasites and the gut microbiota primarily in humans. The purpose of this narrative review is to identify key themes, conflicting findings, research gaps, and propose directions for future research. The methodology was conducted through an iterative, exploratory search and selection process, guided by the author’s expertise in gut microbiome research and parasitology.

### Literature search and study selection

We conducted initial scoping searches across multiple electronic databases, including PubMed, Web of Science, and Google Scholar. Additionally, we complemented manual searches of reference lists to identify relevant studies as needed. Search terms included combinations of the following keywords: “human”, “adult”, “child”, “men”, “women”, “microbiota”, “microbiome”, bacteria”, “flora”, “microbial diversity, “dysbiosis”, “microbial communities”, “parasite”, “intestinal parasites”, “intestinal parasitic infection”, “parasitic infection”, “helminth”, soil-transmitted helminth, protozoa, “Ascaris”, “Trichuris”, “hookworm”, “Giardia”, “Entamoeba”, “Cryptosporidium”, “Blastocystis”. Boolean operators (“AND” and “OR”) were used to combine terms as appropriate. We prioritised studies conducted in humans. Animal and in vitro studies were included selectively to provide a mechanistic context where human data were limited or unavailable. No strict publication date restrictions were applied to ensure inclusion of foundational studies that established key concepts in the field. Only full-text articles published in English were considered.

### Scope and interpretation

The review focuses on qualitative synthesis rather than exhaustive enumeration of all published studies. We organised findings thematically to integrate observational human evidence with experimental mechanistic data. When multiple studies addressed similar research questions, we selected studies based on their relevance, study design, microbiota and parasite characterisation, and their contribution to conceptual understanding, rather than predefined quantitative thresholds. We identified conflicting findings by comparing key studies within and across parasite taxa and host systems. We interpreted divergent results in relation to differences in study design, parasite species, infection intensity, microbiota profiling methodology, and host or environmental context. Human studies were emphasised wherever available. Animal and in vitro studies were included selectively to provide mechanistic insight into parasite-microbiota-host interactions that remain incompletely addressed in human populations.

### Methodological limitations and potential bias

We acknowledge that this narrative approach relies on the author’s judgment and is therefore subject to selection bias. The interpretation and conclusions made in this review inherently reflect the author’s synthesis of the selected literature rather than a comprehensive or quantitatively weighted assessment. While efforts were made to capture representative and influential studies, some relevant publications may have been omitted. Consequently, the conclusions presented reflect the author’s interpretation of the available evidence rather than a definitive or exhaustive summary.

## The Context-Dependent Interplay Between Intestinal Parasite and Gut Microbiota

Interactions between intestinal parasites and the human gut microbiota are not unidirectional. Instead, they reflect a dynamic, bidirectional relationship in which parasite presence and microbial community structure influence one another, with significant consequences for infection outcomes and host health. Intestinal parasites are associated with alterations in the gut environment, including changes in epithelial integrity, immune signalling, and nutrient availability, which, in turn, are linked to shifts in microbial composition and diversity [6]. Conversely, the resident microbiota actively influences parasite establishment, persistence, and pathogenic potential rather than functioning as a passive background community.

Experimental animal models provide evidence for this bidirectionality. In germ-free murine models, infection with the helminth *Heligmosomoides polygyrus* alters parasite gene expression and reduces worm fitness compared with infections in specific-pathogen-free mice, demonstrating that parasite biology depends on the presence of a microbial community [14]. Similarly, both in vitro and animal studies show that bacterial metabolites such as indoles inhibit the growth of protozoa, including *Cryptosporidium parvum* [15]. These findings establish biological plausibility for bidirectional parasite-microbiota interactions, although their direct translation to human infection dynamics remains uncertain.

In human populations, however, studies examining parasite-microbiota associations frequently report heterogeneous and sometimes contradictory findings (Supplementary Table S1). This variability likely reflects the context-dependent nature of these interactions, shaped by multiple interacting factors, including host genetics, diet, environmental exposures, infection intensity, coinfection status, and methodological differences in parasite detection and microbiota profiling [16, 17]. One determinant that is often insufficiently distinguished in the literature is the parasite’s biological class. Helminths and protozoa differ fundamentally in life-cycle complexity, anatomical niches, and host-interaction strategies [2], and therefore may influence the gut microbiota through distinct mechanistic pathways.

Helminths, as larger metazoans with often prolonged residence in the host, are generally associated with broader and more sustained immunological and physical modifications of the gut environment [18]. In contrast, protozoa, which are smaller and often replicate rapidly, tend to exhibit more localised and species-specific associations with the microbiota, with effects strongly influenced by pathogenicity [19, 20]. The following sections synthesise current evidence on the patterns of helminth- and protozoa-associated microbiota, emphasising both shared principles and class-specific differences.

### Helminths

Human studies collectively indicate that helminth infection is associated with changes in gut microbial diversity and composition. Two recent meta-analyses provide quantitative insight into this [18, 21]. These two meta-analyses show a pattern of robust evidence that helminth infections are associated with increased bacterial diversity, as indicated by the overall adjusted effect sizes for Shannon [18, 21] and InvSimpson diversity [18].

Despite this overall trend, substantial heterogeneity exists across individual studies (Table 1). In the meta-analysis by Kupritz et al. [18], which included 17 datasets, seven (41%) found increased alpha diversity in helminth-infected individuals [22–28], four (24%) studies reported higher diversity in uninfected individuals [29–32], and six (35%) studies found no significant differences [33–38]. Similarly, Walusimbi et al. [21] reported that 10 of 15 datasets (66.7%) reported a higher average Shannon diversity in helminth-infected individuals compared to uninfected individuals [24–26, 28, 29, 34, 39], while six datasets reported higher observed richness [25, 34, 39]. However, three studies reported lower Shannon diversity in helminth-positive participants [36, 39, 40], underscoring the variability of alpha diversity responses.

In contrast to alpha diversity, beta diversity changes appear more consistent. Across both meta-analyses, helminth infection significantly contributed to between-sample variation in microbial community composition in 8 of 14 evaluable studies [18]. These shifts were often characterised by altered relative abundances of Firmicutes and Bacteroidetes, reflecting a reorganisation or compression of community structure rather than uniform enrichment or depletion. Because the Firmicutes-Bacteroidetes balance is closely linked to nutrient metabolism and immune development, such compositional shifts may have systemic physiological implications [41].

Several factors contribute to the observed heterogeneity. Age emerged as a strong moderator of alpha diversity effects in both meta-analyses [18, 21]. Across parasite-microbiota interaction studies in humans, participants’ ages ranged from children to adults (Supplementary Table S1), with children disproportionately represented due to their higher exposure risk in endemic settings [42]. Although gut microbial communities broadly stabilise by early childhood, age-related plasticity persists and may amplify parasite-associated effects [43]. For example, helminth infection was associated with a significant shift in enterotype from *a Bacteroides*-rich to a *Prevotella*-rich community in a cross-sectional study of children in Colombia [30]. Diet typically influences enterotypes over the long term [44], and this study highlights helminths’ ability to induce enterotype shifts not generally seen with short-term dietary fluctuations. These findings are also corroborated by a study in a mother-child pair in Tanzania with chronic *T. trichiura* infections [45]. These observations suggest that helminths may exert a powerful influence on age groups with greater microbiota plasticity. Further study is needed with age as a standardizing factor.

Diet represents another critical but inconsistently assessed factor. Diet is a primary determinant of gut microbiota composition [46], yet only a minority of helminth-microbiota studies explicitly account for dietary intake [22, 30, 45, 47–49]. Dietary substrates influence both parasite survival and microbial production of short-chain fatty acids (SCFAs), which play key roles in gut barrier integrity and immune regulation [50]. Given substantial geographic variation in dietary patterns across endemic regions, unmeasured dietary differences are likely to confound comparisons between populations. Notably, in Malaysian indigenous communities where dietary intake was relatively homogeneous, *T. trichiura* infection remained associated with microbiota variation after accounting for diet, suggesting that helminth effects can occur independently of dietary background [48]. Nonetheless, larger studies with standardised dietary assessment are needed to confirm these findings.

Biological differences among helminth species further complicate interpretation. Whipworms (*T. trichiura*), roundworms (*A. lumbricoides*), and hookworms differ in anatomical niche, feeding behaviour, and host interaction strategies, all of which may differentially shape microbial communities. Most human studies rely on faecal sampling for microbiota profiling due to feasibility constraints, potentially biasing detection toward colon-dwelling parasites. *T. trichiura* resides in the cecum and proximal colon, regions of high microbial density and prolonged host–microbiota contact, potentially explaining the more consistent faecal microbiota alterations reported in trichuriasis [48, 49]. In contrast, hookworms attach to the duodenal mucosa, a region with lower microbial density, and their effects may be localized and diluted in faecal samples. Accordingly, longitudinal human studies of *N. americanus* infection have reported minimal or no changes in overall microbial diversity [47, 51, 52]. However, these comparisons should be interpreted cautiously, given limited sample sizes and methodological variability. Together, these findings highlight a key limitation of faecal sampling, which may fail to capture microbiota changes in the small intestine. While biopsy-based approaches offer greater spatial resolution, their invasive nature limits applicability in human studies.

More broadly, the heterogeneous microbiota signatures reported across helminth-infected populations likely reflect a convergence of biological and methodological factors rather than factual inconsistency in parasite effects. Geographic variation in microbiota responses may arise from differences in host genetic background, parasite strain diversity, and baseline microbiota composition shaped by long-term dietary patterns, particularly fibre intake and availability of fermentable substrates [17, 53]. In addition, undetected or unreported coinfections, a common occurrence in endemic settings, may confound attribution of microbiota changes to single helminth species. Methodological heterogeneity across studies, including variation in DNA extraction protocols, sequencing depth, target regions of the 16 S rRNA gene, and taxonomic resolution, further complicates cross-study comparisons (Supplementary Table S1). Finally, differences in infection chronicity and intensity, which are often inferred from single time-point diagnostics or egg counts, may obscure biologically meaningful gradients in microbiota responses. Available evidence suggests that moderate to heavy soil-transmitted helminth infections are more consistently associated with increased alpha diversity and compositional shifts, whereas low-intensity infections may have minimal detectable effects [25, 54]. Together, these factors suggest that mixed findings across populations reflect context-dependent parasite–microbiota interactions rather than contradictory evidence. These considerations underscore the importance of interpreting patterns of helminth-associated microbiota within their ecological, methodological, and epidemiological contexts.

Finally, the predominance of observational study designs limits causal inference. The heterogeneity observed across the literature likely reflects variation in geography, host factors, parasite species, infection intensity, coinfection patterns, sample size, and methodological approaches. Consequently, it remains difficult to distinguish whether observed microbiota differences reflect helminth-driven ecosystem remodelling, pre-existing microbial configurations that influence susceptibility to infection, or shared environmental and socioeconomic determinants. Current human evidence therefore supports association rather than causation.

### Protozoa

Unlike helminths, protozoa often induce more consistent and pronounced changes in the gut microbiota, generally associated with dysbiosis, though the consistency of this association varies by species and context (Supplementary Table S1). A key methodological consideration is that variation in diagnostic approaches from microscopy to PCR-based methods can affect the apparent prevalence and accurate identification of protozoan species, limiting direct comparisons across studies and potentially contributing to inconsistent findings. The impact on microbial diversity is closely correlated with protozoan pathogenicity, with pathogenic species typically associated with decreased diversity [20] and commensal species with increased diversity [55].

#### Pathogenic Protozoa

Infections with pathogenic protozoa such as *G. duodenalis*, *E. histolytica*, and *Cryptosporidium* spp. are generally associated with dysbiosis in most studies, though inconsistencies have been documented. However, findings across studies reveal significant heterogeneity, reflecting differences in parasite species, host health status, and methodological approaches.

*G. duodenalis* infection presents particularly heterogeneous findings. Some studies report significantly reduced alpha diversity in infected individuals, accompanied by taxonomic shifts such as decreased Firmicutes and increased *Prevotella* abundance [27, 30]. The degree of microbial alteration may correlate with parasite burden [27]. However, other well-designed studies have found either no significant differences in diversity [56] or even increased diversity [57] associated with giardiasis. These discrepancies may stem from variations in study populations such as symptomatic versus asymptomatic individuals, age groups, geographical settings, or methodological factors, including different 16 S rRNA gene regions targeted for profiling (Supplementary Table S1).

Studies on *E. histolytica* similarly yield inconsistent findings on diversity, with reports of increased [58], decreased [59], or unchanged [60] alpha diversity in infected individuals. Notably, the clinical context appears to influence these associations. Studies involving patients with symptomatic amoebiasis or diarrhoea tend to report more pronounced dysbiosis and reduced diversity [59, 61, 62], while research on asymptomatic carriers may show minimal diversity changes despite compositional shifts [60].

Meanwhile, evidence for *Cryptosporidium* spp. is more limited and comes from preliminary investigations in clinical case series, which can offer valuable insights despite limited sample sizes. In patients with underlying immunodeficiencies or malignancies, cryptosporidiosis has been linked to reduced alpha diversity and the expansion of pathobiont-associated taxa, such as *Enterobacteriaceae* [63, 64]. As these findings are based on small, cross-sectional case studies, they should be interpreted as hypothesis-generating rather than definitive, highlighting the need for larger cohort studies to validate these associations in broader populations. Similarly, microsporidia infections in cancer patients are associated with decreased diversity and increased pathobionts [65]. These findings in vulnerable populations highlight how immune status modulates protozoa-microbiota interactions.

The heterogeneity observed across studies of pathogenic protozoa likely reflects multiple factors, including parasite pathogenicity and burden, differences in host health status and immune competence, geographical and dietary influences, and methodological disparities in parasite detection and microbiota profiling. Future study would benefit from standardised diagnostic approaches, consistent reporting of clinical metadata, and larger, longitudinal studies to better disentangle these complex interactions.

#### Commensal Protozoa

The role of commensal protozoa, such as *Blastocystis* spp. and non-pathogenic *Entamoeba* spp., in shaping the gut microbiota is an area of active research and debate. Colonisation by *Blastocystis* spp. is frequently associated with increased bacterial diversity and a reduction in pathobiont-associated taxa, such as *Enterobacteriaceae* and *Proteobacteria* [66–71]. However, adverse associations have also been reported [68, 72]. This contradiction appears to be subtype-specific. Subtypes ST1, ST3, and ST4 are often associated with beneficial bacteria and are considered markers of a healthy gut [73–77], whereas subtype ST7 has demonstrated pathogenic potential in model systems [78]. Resolving this controversy will require standardised subtype identification protocols, subtype-specific intervention studies, and clear criteria for pathogenicity assessment that integrate colonisation frequency, abundance, and correlation with clinical symptoms.

Similarly, colonisation by non-pathogenic *Entamoeba* spp. (e.g., *Entamoeba coli*) is also associated with increased microbial diversity, characterised by a higher Firmicutes-to-Bacteroides ratio [58, 79]. In healthy children, this is accompanied by an enrichment of beneficial bacteria, such as *Akkermansia* and butyrate-producing *Coprococcus* [80, 81]. Research on other commensal protozoa, such as *Dientamoeba fragilis*, is limited. Preliminary evidence from a single cross-sectional study suggests that *D. fragilis*-positive children have an altered abundance of 16 bacterial genera, including beneficial *Victivallis*, *Oscillibacter*, and *Coprococcus*, and a decrease in *Flavonifractor* [82]. These initial findings warrant further investigation in larger, diverse populations.

## Parasite Burden, Clearance, and Antiparasitic Drugs as Determinants of Microbiota Dynamics

Variability in gut microbiota responses to parasitic infections reflects not only differences between parasite species, but also differences in infection intensity, chronicity, and treatment. Low or acute infections may cause subtle, reversible shifts, whereas heavy or chronic infections often drive more profound and persistent dysbiosis. In addition, antiparasitic drugs can exert direct antimicrobial effects on the resident microbiota, confounding post-clearance microbial patterns. However, a major challenge across studies is the lack of standardised definitions of parasite burden, which limits direct comparison and synthesis. This section integrates evidence on infection intensity, clearance, and treatment effects to clarify their combined influence on parasite–microbiota dynamics.

### Helminths

The relationship between helminth burden and microbiota alteration is dose-dependent, but quantifying this relationship is challenged by inconsistent definitions of ‘low’ and ‘high’ burden across studies. For STHs, the WHO provides intensity thresholds that classify light-, moderate-, or heavy-intensity [83]. However, applied thresholds in microbiota research are rarely reported without quantitative EPG (number of parasite eggs per gram) data. This methodological heterogeneity complicates synthesis and must be considered when interpreting burden-dependent effects in future studies.

Low-burden helminth infections, often acute or low-intensity, are typically associated with minimal epithelial disruption and modest, transient changes in the microbiota. For example, an experimental low-dose *N. americanus* infection in humans did not significantly alter overall microbial diversity in the acute phase [47, 51]. According to a study of acute helminth infections in equine youngstock, the clearance of these infections is often accompanied by a shift in the gut microbiota toward a pre-infection configuration, defined here as a partial or near-complete restoration of community structure and relative taxonomic abundance compared with the infected state [84].

In contrast, high-burden or chronic helminth infections are more consistently associated with pronounced and sustained alterations in the microbiota [21]. These infections establish a persistent immunomodulatory environment characterised by Th2 polarization and regulatory immune pathways that promote parasite persistence while limiting host tissue damage [85, 86]. Higher parasite loads are therefore associated with stronger and more enduring microbial shifts [87, 88]. For example, in three cross-sectional studies in Sri Lanka, Thailand, and Tanzania, higher egg counts of STH correlated with reduced microbial diversity and greater compositional restructuring [33, 49, 54]. This likely reflects a stronger, more sustained immunomodulatory effect and a greater physical perturbation of the gut environment.

**Fig. 1:**
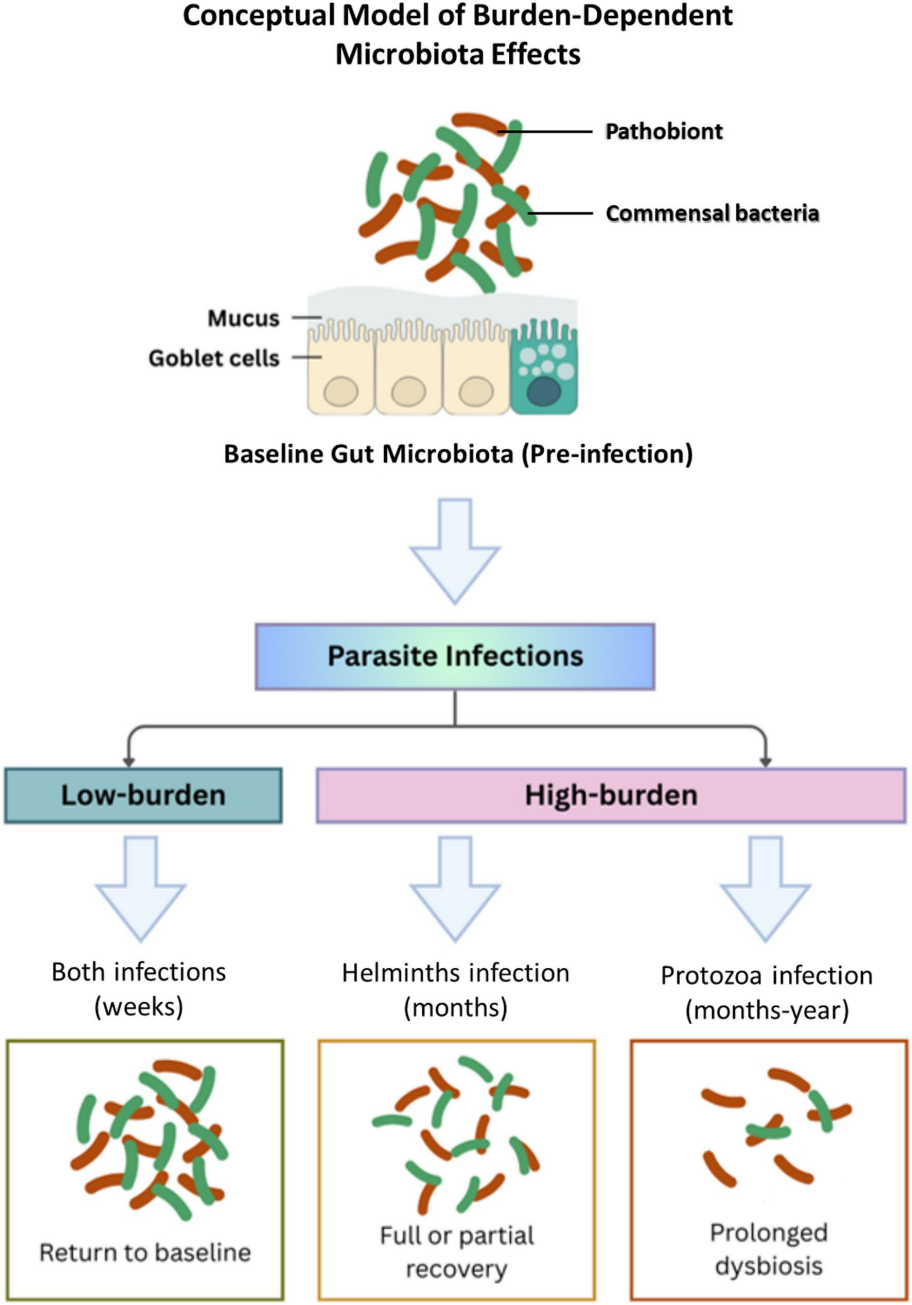
This model illustrates theoretically predicted patterns of microbiota change and recovery based on the synthesis of the reviewed literature. The timelines and outcomes represent hypothesised relationships. Empirical data are required to validate these predictions and define quantitative thresholds

The microbiota’s ability to recover following helminth clearance is not uniform. It depends on whether the clearance is immune-mediated (natural or resolves on its own) or achieved with anthelmintic drugs. In low-burden helminth infection, the host resolves itself through Th2-polarised immune responses [85, 86]. However, human data on natural clearance are scarce due to the difficulty of long-term observation in untreated cohorts. In an animal model study in youngstock with acute helminth infections, short-duration, low-burden helminths, the microbiota exhibits high resilience, eventually returning to a pre-infection configuration [84] (Fig. 1). In contrast, chronic infections may follow more complex recovery trajectories. For example, in an experimental murine model infected with chronic *Trichuris muris*, the microbiota does not revert but undergoes successional recovery before reaching a state similar to that of an uninfected state [89].

High-burden infections are usually cleared via anthelmintic treatment. Unlike gradual immune-mediated clearance, deworming with drugs such as albendazole rapidly removes the parasite. However, based on cross-sectional and longitudinal studies in two endemic regions, Indonesia and Liberia, post-treatment microbiota profiles in humans often resemble the pre-treatment infected state more than the uninfected state, suggesting that helminth clearance alone does not immediately restore microbial composition [25]. The direct antibacterial activity of anthelmintics further complicates recovery. Albendazole, for example, has broad-spectrum antimicrobial properties, meaning observed post-treatment changes likely reflect a combination of parasite removal and drug effects [34].

Evidence for drug-specific effects is mixed. Some studies found albendazole treatment affected bacterial communities regardless of infection status [36], while others showed no significant changes in microbiota in uninfected individuals taking the drug [34]. In the study by Martin et al. [34], albendazole had minimal effects on the gut microbiota. Instead, significant differences in microbial composition between the albendazole and placebo groups were observed in those who remained infected post-treatment. This reflects the interaction between the gut ecosystem and helminth species such as *T. trichiura*, which makes anthelmintics like albendazole less effective against *T. trichiura*.

Currently, the direct impact of deworming treatment on the human gut microbiome remains uncertain, as some studies have found no effect [29, 90], while others have observed changes [17, 22, 36]. Disentangling these effects requires study designs that include mock-treated controls, longitudinal monitoring in naturally reinfecting populations, or comparison across drugs with differing antimicrobial spectra. At present, variability in drug type, dosage, and treatment frequency limits generalisation across studies.

### Protozoa

In contrast to helminths, protozoan parasites interact more directly with the gut epithelium and luminal microbial community through mechanisms such as epithelial attachment, nutrient competition, cytotoxicity, and localised inflammatory signalling. As a result, parasite burden and clearance exert more immediate and often more disruptive effects on microbiota structure and function. However, compared with helminths, longitudinal human studies examining protozoa–microbiota dynamics remain limited, constraining definitive conclusions regarding recovery trajectories following clearance. Protozoan burden is typically inferred from symptom severity (higher intensity correlates with more severe symptoms), parasite detection frequency, or molecular load, rather than standardised quantitative thresholds, which complicates cross-study comparisons.

Low-burden protozoa infections tend to cause an immediate, localised shift in bacterial composition and function that often correlates with acute symptoms, in contrast to the broad, large-scale dysbiosis seen in high-burden or invasive infections [91, 92]. The direction of these changes depends on whether the protozoa are commensal or pathogenic. For instance, commensal protozoa like *Blastocystis* are often associated with higher microbial diversity, even at low burdens [67, 93]. Conversely, low-burden pathogenic protozoa (e.g., *G. duodenalis*, *E. histolytica*, and *Cryptosporidium* spp.) can shift communities toward a loss of beneficial taxa or induce functional changes, even in asymptomatic cases. For instance, a low-burden *Giardia* infection in children can reduce *Lactobacillus* and *Bifidobacterium* without causing diarrhoea [92, 94]. This is likely due to localised nutrient competition, low-level predation, and subtle immune signalling.

In contrast to these subtle modulatory effects, high-burden protozoan infections, particularly with pathogenic protozoa, often lead to strong mucosal damage and robust inflammation. This results in major metabolic disturbances, greater loss of beneficial SCFA-producing bacteria, and the bloom of pathobionts [95, 96]. As a result, these infections often cause a large reduction in microbial diversity, especially when symptomatic. For example, a high *E. histolytica* burden in children was associated with the expansion of *P. copri* and diarrhoea [97]. In moderate to severe cryptosporidiosis, there is a reduction in alpha-diversity and an enrichment of *Enterococcus* spp., whereas milder manifestations of the disease show decreases in beneficial bacteria such as *Bifidobacterium*, *Gemmiger*, and *Blautia* [64]. This indicates a loss of microbial resilience and a shift toward a less stable, more pro-inflammatory state.

Evidence regarding microbiota recovery following protozoan clearance remains limited but suggests that restoration to a pre-infection state may be incomplete, particularly after high-burden or symptomatic infections (Fig. 1). Available data indicate that protozoan clearance may be followed by persistent alterations in microbiota composition and function; however, well-designed longitudinal studies are needed to determine the frequency, duration, and reversibility of these changes. A common and debilitating example is the post-giardiasis syndrome, which can persist after the parasite is eliminated and presents with symptoms similar to those of irritable bowel [98, 99]. This phenomenon is thought to result from persistent gut barrier dysfunction, inflammation, and immune dysregulation induced by the initial pathogenic protozoa, leaving the gut unable to recover its diversity and functional balance. These processes may collectively impair microbiota resilience and delay functional recovery, a topic explored further in Sect.  6.

Additionally, the use of anti-protozoal drugs, such as metronidazole, further complicates recovery. These drugs have a broad-spectrum antimicrobial effect and are known to cause significant and prolonged dysbiosis [100]. This can hinder the microbiota’s ability to revert to a healthy state and exacerbate symptoms. For example, eradicating protozoa such as *Blastocystis* spp. can be challenging, with conventional treatments often failing to achieve complete clearance [101]. Other antiprotozoals, including nitazoxanide, paromomycin, and furazolidone, differ substantially in pharmacokinetics and antimicrobial spectra. However, their specific effects on the gut microbiota remain largely uncharacterized. Distinguishing parasite-driven effects from drug-induced perturbations, therefore, represents a major methodological challenge in this field. Given these complexities, although general studies of gut microbiota dynamics or drug-induced changes exist, detailed, protozoa-specific recovery data remain notably scarce. This limitation severely hinders our ability to understand the precise trajectory of microbial changes, the mechanisms of recovery or persistence of dysbiosis, and the long-term health consequences of these infections.

Taken together, current evidence supports a model in which protozoan burden and clearance exert strong, often rapid effects on gut microbiota composition, with pathogenic species and high burdens posing the greatest risk for persistent dysbiosis. However, the scarcity of longitudinal, protozoa-specific microbiome studies limits mechanistic resolution and underscores the need for carefully controlled investigations that disentangle parasite burden, host response, and treatment effects.

## Polyparasitism: A Complex Ecological Outcome

**Fig. 2:**
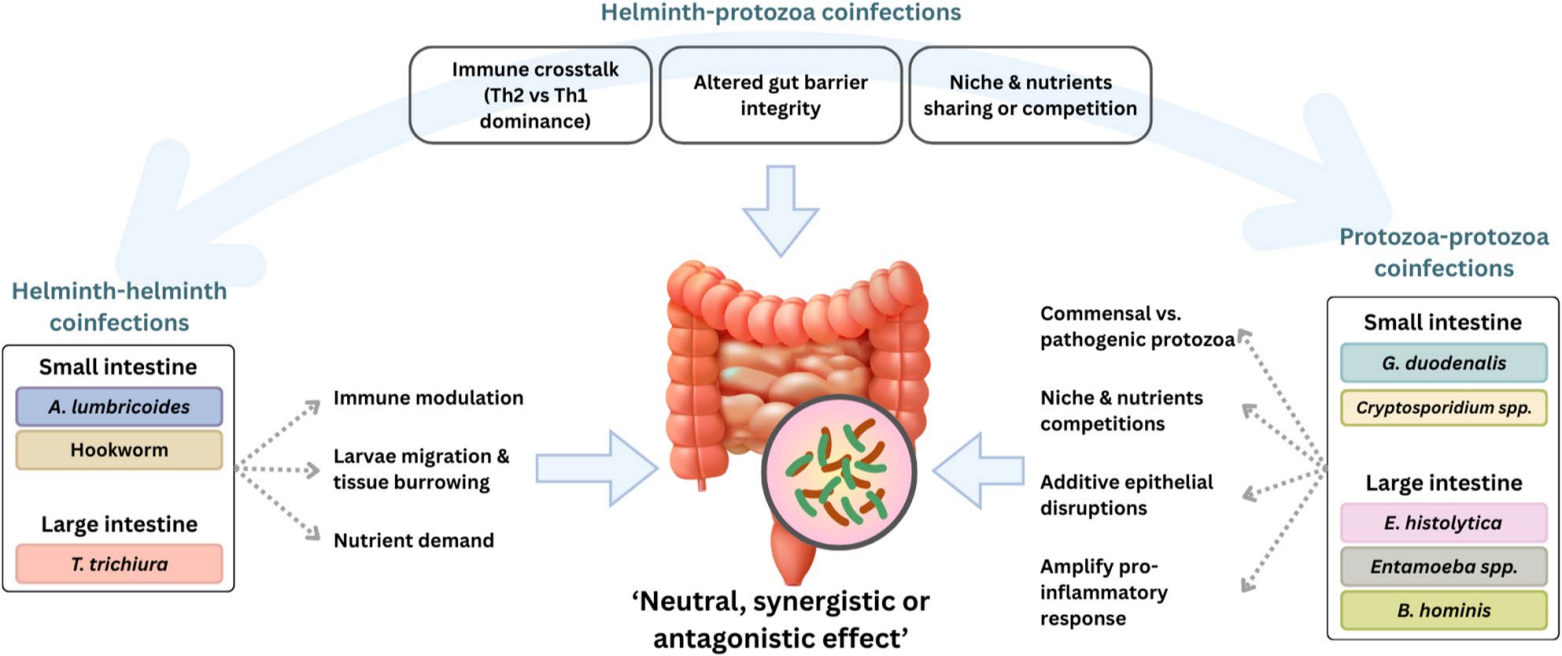
Conceptual Framework of the dynamics of polyparasitism on the gut microbiota

A current aspect in parasite-microbiota interactions that is rarely discussed in literature is polyparasitism, the concurrent infection of a single host by multiple parasites, which represents the epidemiological norm in endemic regions, driven by shared risk factors like poverty, poor sanitation, and high pathogen exposure [102]. While the prevalence of polyparasitism is well-documented [26, 102–106], its impact on the gut microbiota is poorly characterised, and most research has failed to address the complex dynamics of polyparasitism that reflect real-world conditions. Moreover, multiple shared risk factors that independently influence microbiota composition make it difficult to disentangle true parasite-parasite interaction effects from confounding. Addressing this requires either (a) multivariate analyses adjusting for socioeconomic, environmental, and dietary covariates, (b) studies conducted in more homogeneous populations, or (c) instrumental variable approaches. The interactions can be neutral, synergistic, or antagonistic (Fig. 2). This section reviews emerging evidence for these interaction types while explicitly acknowledging the substantial methodological challenges that limit definitive conclusions.

### Helminth-Helminth Coinfections

Helminth-helminth coinfections remain poorly understood, as current studies do not provide sufficient evidence to identify clear patterns or develop predictive models. The available research shows mixed results. Some robust studies link polyparasitism to higher microbial diversity [22, 25, 26, 90], whereas other studies find no significant association [33, 36, 107]. This variability stems from the distinct immunological and physical niches occupied by different helminths (e.g., luminal *T. trichiura* versus mucosal hookworms), which may lead to independent or spatially segregated effects on the gut ecosystem. Furthermore, the nutrient demand of one helminth may affect others, potentially impacting the microbiota itself.

Most of these studies compare coinfected individuals with helminths to uninfected controls rather than to single-species infections, limiting inference about interaction effects. A study in Ecuadorian children found that coinfection with *T. trichiura* and *A. lumbricoides* was associated with reduced bacterial diversity and decreased Clostridia compared with *T. trichiura* infection alone, suggesting that *A. lumbricoides* may exert a distinct influence [29]. However, limited sample sizes and confounding factors preclude generalisation. Elucidating the rules governing these interactions represents a clear priority for future research.

### Helminth-Protozoa Coinfections

Helminth-protozoa coinfections introduce additional complexity due to the contrasting life histories of the parasites. Helminths typically establish chronic infections characterised by immunoregulatory responses, whereas pathogenic protozoa often induce localised epithelial damage and acute inflammation. Their interactions demonstrate clear non-additive effects. For example, children in Colombia coinfected with *Giardia* and helminths exhibited the lowest alpha diversity and a marked shift from a *Bacteroides*-dominant to a *Prevotella*-dominant enterotype compared to infections with just protozoa or healthy individuals [30]. The study also revealed a striking shift from a *Bacteroides*-dominated type to a *Prevotella*-dominated type in children with helminth and *Giardia* coinfections, a change that’s typically difficult to achieve in humans. Reinforcing this complexity, another study by Mejia et al. [27] noted that while helminth-only infections increased microbial diversity, helminth-*Giardia* coinfections decreased it. They also observed that *Prevotella* abundance decreased in helminth-only groups but increased in coinfected groups.

Other examples that reflect these dynamics, Iebba et al. [108] reported that children coinfected with *Giardia duodenalis*, *Entamoeba* spp., and helminths displayed microbiota profiles distinct from those with protozoan infections alone, including differences in the relative abundance of *Bacteroides* and *Prevotella*. A plausible explanation for these observations, helminth-induced immunoregulatory pathways may dampen inflammation-driven dysbiosis caused by protozoa, while protozoan-induced epithelial disruption may override or modify helminth-associated microbiota signatures. As a result, helminth-protozoa coinfections often yield microbiota patterns that differ qualitatively from those observed in single infections, rather than representing simple additive effects.

### Protozoa-Protozoa Coinfections

Coinfections between different protozoa species can also significantly influence microbial diversity and composition, with distinct outcomes emerging between pathogenic and potentially commensal species. A large-scale study in Guinea-Bissau revealed that protozoan infections, including common species like *Entamoeba* spp. and *G. duodenalis*, significantly reshaped the entire microbial community [109], underscoring that the presence of multiple protozoa can profoundly modify the gut environment. In a cross-sectional study in Italy involving diarrheal patients with single infections with *Blastocystis* spp., *Dientamoeba fragilis*, *G. duodenalis*, and *C. parvum*, or coinfections with protozoa, researchers observed distinct species-specific bacterial profiles, indicating that different protozoa are associated with unique gut bacterial signatures [110].

Conversely, not all protozoan coinfections are necessarily detrimental. A preliminary study on coinfections of *Blastocystis* and *Entamoeba* spp. found an association with a healthier gut environment, characterised by a higher ratio of the beneficial butyrate-producer *Faecalibacterium prausnitzii* to pathobionts such as *E. coli* [108]. The increased presence of *F. prausnitzii*, a key producer of butyrate, is particularly significant as butyrate is a crucial short-chain fatty acid that provides energy to colonocytes, strengthens the gut barrier, and exhibits anti-inflammatory properties [111, 112]. This suggests that in certain contexts, the presence of a commensal protozoan might buffer the negative impacts of pathogenic coinfections or foster a more resilient microbial community, though this area warrants further investigation.

### Methodological Challenges in Polyparasitism Research

Polyparasitism is common in many endemic settings and represents both a major confounder and a biologically relevant context for interpreting parasite-microbiota associations [26]. Failure to account for concurrent infections likely contributes to the heterogeneous and sometimes contradictory findings reported across studies (Supplementary Table S1). However, the primary analytical challenge in polyparasitism research therefore does not stem from the rarity of coinfection, but from the diversity of parasite combinations and infection states observed within populations. As the number of co-circulating parasite species increases, study populations become fragmented across multiple biologically distinct coinfection profiles, limiting statistical power to detect interaction effects for specific parasite pairs or groups. This challenge is compounded when infection intensity, chronicity, and host factors are considered simultaneously. In addition, testing multiple parasite combinations raises concerns about multiple comparisons and the risk of false-positive findings. Consequently, robust inference of parasite-parasite-microbiota interactions requires large sample sizes, targeted study designs, or coordinated analyses across cohorts. Given these constraints, conclusions regarding interaction effects should be interpreted cautiously.

## Mechanistic Basis of Parasite-Microbiota Interactions

**Fig. 3:**
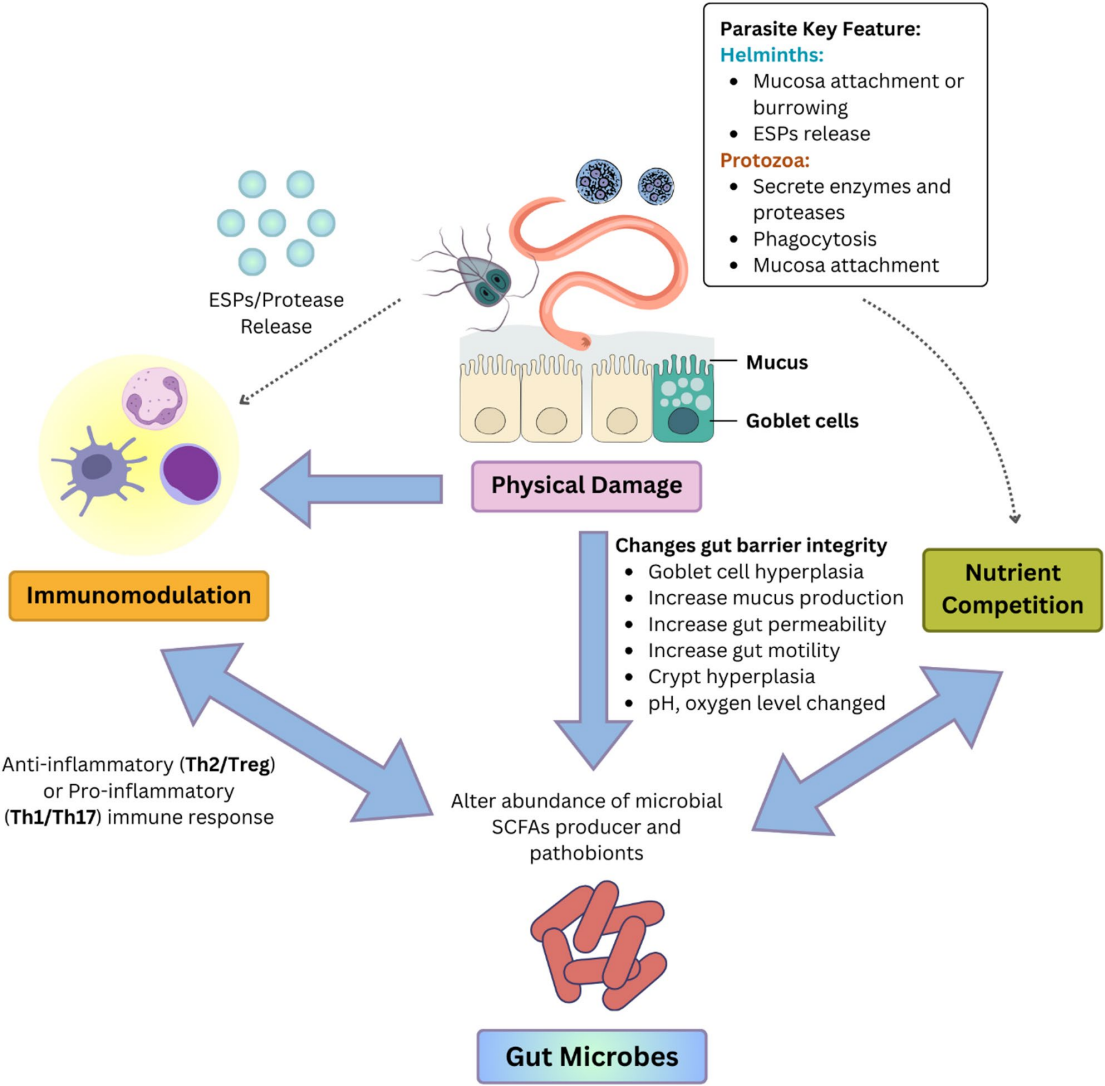
Key mechanism of helminths and protozoa interactions with the gut microbiota

In this review, the mechanisms underlying parasite-microbiota interactions were compared across helminths and protozoa. A critical caveat is that direct mechanistic evidence of gut microbiota interactions from human studies is limited. Therefore, the following discussion necessarily integrates clinical observations with findings from animal models and in vitro studies to construct plausible mechanistic frameworks. While these experimental models provide indispensable and detailed insights, their limitations, including species-specific biology of model parasites and differences in host immunity and diet compared to humans, must be considered when extrapolating to human physiology. In this context, helminths and protozoa employ distinct strategies for survival, leading to distinct physiological and immunological effects (Fig. 3). Helminths are the master regulators of the immune system. Hence, they tend to exert their influence on the gut microbiota through more indirect and long-term immunomodulatory effects [18]. In contrast, protozoa often have more direct and immediate interactions with the gut microbiota, which can significantly influence their virulence and host pathology [19].

### Helminths

#### Immune modulation

Human helminth infections elicit complex immune responses. While immune responses in humans are generally more nuanced and less polarised than those observed in experimental settings, this review synthesised a simplified immunological framework to describe the dominant pathways by which helminths influence host physiology and gut microbiota composition. Within this framework, helminths predominantly induce Type 2 (Th2) immune response following tissue damage caused by helminth migration and burrowing [85, 86]. This response is characterised by the production of cytokines such as interleukin-3 (IL-3), IL-4, IL-5, IL-9, and IL-13, and by the activation of various immune cells, including eosinophils, basophils, and mast cells [86, 113]. The Th2 immune response is thought to be necessary to expel helminths from hosts [86] and creates a broadly anti-inflammatory milieu within the host, crucial for wound healing and tissue repair [85].

However, helminths can subvert this response to prevent a damaging, overzealous inflammatory reaction that could harm both the host and the parasite. They do this by inducing a powerful regulatory counter-response that dampens the inflammatory arm of immunity. A key component of this counter-response is the expansion and activation of regulatory T cells (Tregs) [114]. These cells are crucial for maintaining immune tolerance and preventing autoimmunity, and their induction by helminth parasites helps dampen the inflammatory effects of the Th2 response. This is achieved by Tregs producing high levels of anti-inflammatory cytokines, most notably IL-10 and transforming growth factor-β (TGF-β), which suppress the proliferation and function of effector T cells [115].

Helminths also have the key ability to manipulate host immunity and ensure their survival by releasing a diverse array of excretory-secretory products (ESPs) [116–118]. The ESPs comprised peptides, lipids, proteins, carbohydrates, organic acids, and nucleic acids [119]. These molecules can directly interact with host immune cells, interfere with signalling pathways (e.g., by targeting pattern recognition receptors like Toll-like Receptors (TLRs)), or mimic host’s immunologically relevant proteins or miRNAs that target the host’s gene expression [120, 121], contributing to the overall immunomodulatory effect that shapes the gut environment and, consequently, the microbiota.

#### Physical Alterations to the Gut Environment

Helminths employ various species-specific strategies to attach or migrate through the gut wall, causing physical damage. For instance, hookworms use their buccal capsules to attach to the gut mucosa, resulting in damage and bleeding [122]. Similarly, the larval stage of human helminths, such as *A. lumbricoides*, migrates through the gut mucosa, creating microscopic tunnels that disrupt the epithelial barrier [123]. This direct damage can result in villous atrophy, a flattening of the gut’s villi [124]. In response to this damage, the host develops crypt hyperplasia, an increase in crypt size and cell proliferation, as the host attempts to repair the damaged epithelium by increasing cell turnover to shed helminths [125].

The tissue damage also triggers the Th2 immune response [85], characterised by cytokines IL-4 and IL-13, which initiate the “weep and sweep” response, a series of physiological changes designed to expel the helminths [125]. This response stimulates goblet cell hyperplasia, an increase in mucus-secreting cells, and upregulation of mucin sialylation [126, 127], resulting in hypersecretion of a dense, viscous mucus that can trap and physically dislodge the worms from their luminal niche while protecting the underlying epithelium [128]. This robust mucus layer not only supports beneficial mucus-residing bacterial species but also limits pathogen access. For example, in studies with mice infected by the murine roundworm *Heligmosomoides polygyrus*, there was a notable rise in colonic goblet cells and sialylation, primarily driven by IL-13. This change was accompanied by an increase in the abundance of *Ruminococcus gnavus*, a bacterium capable of utilising sialic acid as an energy source [127]. Similarly, helminth-infected individuals show increased abundance of *Akkermansia muciniphila*, likely due to increased mucin production driven by both the helminth and the host [33].

Simultaneously, Th2 immune responses enhance gut epithelial permeability, facilitating the transport of antibodies and immune cells into the gut lumen [125, 129]. The process itself involves mast cells that degrade tight junction proteins such as occludin, alter the expression of adherens junction proteins such as E-cadherin [130, 131], and upregulate the pore-forming tight junction protein claudin-2, which weakens cell-cell adhesion and increases the flow of water and ions across the epithelial lining [132]. This heightened permeability leads to a significant shift in the gut microbiota, often marked by an expansion of *Bacteroidetes* and a decline in *Firmicutes* and *Lactobacillales* [133]. The Th2 cytokine IL-13 and mast cell proteases also increase intestinal muscle contraction and the flow of fluids into the lumen to flush out helminths [134, 135]. These changes in peristalsis and fluid dynamics directly influence the gut’s microbial composition, favouring species better adapted to the new conditions.

#### Nutrient Competitions

Helminths require a steady supply of host-derived nutrients such as carbohydrates, amino acids, lipids, and essential vitamins for growth and reproduction. This puts them in direct competition with the host’s gut microbiota, which also relies on these same resources for energy production [24]. For example, a study in an animal model of *Ascaris suum* in pigs showed that the worms can absorb amino acids and glucose, and that their secreted products inhibit intestinal nutrient uptake [136]. This can often exacerbate malnutrition with compounding stunting and underdevelopment, especially in children [137]. Additionally, helminths secrete enzymes to aid in nutrient acquisition or tissue penetration [138–140]. These enzymes can directly break down complex host dietary components in the gut lumen, creating substrates that are then utilised by specific members of the microbiota. This can effectively “feed” certain bacterial populations, leading to their proliferation. For example, in humans infected with *Strongyloides stercoralis*, elevated levels of amino acids and low levels of short-chain fatty acids (SCFAs) were observed in faecal metabolomes. Its faecal microbial profiles showed increases in Clostridia species, known to be adept at amino acid fermentation, and in lactic acid-producing bacteria, *Leuconostocaceae* [24].

### Protozoa

#### Direct Interactions with the Gut Microbiota

Key aspects of protozoan infections are their direct engagement with the microbiota. These interactions often involve competition for niche and resources, direct predation, and metabolic exchange with the gut microbiota. For example, *Giardia* infection scavenges lipids and excretes novel end products of lipid metabolism [141, 142], enriching metabolically flexible taxa like Gammaproteobacteria (e.g., *Acinetobacter*) while causing a decline in obligate anaerobes like *Firmicutes* and Melainabacteria [142–144]. Similarly, *Giardia* can use arginine for energy production, depleting this amino acid and producing nitrogen-rich byproducts that may support bacteria such as Betaproteobacteria (e.g., *Rhodocyclaceae*) [142].

Some protozoa, particularly pathogenic amoebae like *E. histolytica* trophozoites, exhibit selective phagocytosis, engulfing beneficial bacteria, especially members of the *Lactobacillales*,* Bifidobacteriales*,* Erysipelotrichales*, and *Clostridiales*, as observed in an in vitro study [91]. This selective predation can significantly deplete health-promoting taxa that support gut barrier function and anti-inflammatory signalling, thereby inducing a dysbiosis. Metabolic exchange also represents a crucial direct interaction. Based on in vitro studies, some protozoa, such as *E. histolytica*, can salvage bacterial metabolites, including queuine, a unique molecule produced by the gut microbiota and released upon bacterial lysis. This salvage queuine can regulate the protozoa’s transcriptome, influencing its resistance to oxidative stress and downregulating virulence genes [145]. Conversely, the gut microbiota can produce vital metabolites such as SCFAs, which are indispensable for maintaining gut homeostasis, regulating immune response, and influencing protozoan survival [55].

#### Indirect Interactions via Immunomodulation

Protozoa can elicit a wide spectrum of host immune responses, ranging from pro-inflammatory to anti-inflammatory, depending on their pathogenicity. Commensal protozoa can polarise the T cell response towards a Th2-dominated profile, leading to downregulation of tumour necrosis factor (TNF) and a decrease in the pro-inflammatory response, often resulting in an enrichment of SCFA producers and a reduction in pathobionts [55]. In contrast, pathogenic protozoa often provoke host pro-inflammatory cascades, initiating innate and adaptive immune responses involving TLRs, immunoglobulins (IgM, IgG, IgA), and immune cells [146, 147]. However, these protozoa often exhibit a sophisticated strategy of immune modulation by directly cleaving host-produced chemokines and cytokines, thereby dampening immune responses and evading adaptive immunity to ensure their survival. For example, *G. duodenalis* triggers a host immune response dominated by Th17 and IgA production for the clearance of protozoa [148, 149]. However, *Giardia* can dampen and evade host defenses by reducing arginine availability through arginine deiminase (ADI), impairing nitric oxide (NO) production and epithelial cell proliferation to ensure its survival [150]. Conversely, *E. histolytica*, through expression of Gal/GalNAc lectin to facilitate epithelial invasion and tissue destruction [151], simultaneously elicits secretory IgA response and activates innate immunity via epithelial cells, neutrophils, and macrophages for parasite clearance [152]. However, *E. histolytica* recruits various strategies to evade the immune system, involving gut barrier disruption by hydrolases (e.g., cysteine proteases, glycosidases) [153, 154], cell surface decorations (e.g., glycosylphosphatidylinositol (GPI)-anchored proteins, surface receptor capping), and phagocytosis or trogocytosis of immune cells [155, 156].

#### Alteration in Gut Barrier Function

Pathogenic protozoa are highly effective at compromising the gut environment by altering the epithelial barrier and disrupting the mucus layer, collectively reshaping the local gut microbiota. A primary mechanism is the direct disruption of the gut epithelial barrier. For example, *G. duodenalis* uses a ventral adhesive disc to attach to the brush border of enterocytes. This leads to villous atrophy, which reduces the surface area available for bacterial colonisation and nutrient absorption, thereby shifting the microbial composition toward new niches [157]. Additionally, protozoa can disrupt the mucus layer through secreted molecules. *E. histolytica*, for instance, secretes cysteine proteases that degrade mucin and tight junction proteins [153, 158]. This creates a “leaky gut,” compromising the barrier’s integrity and increasing gut permeability, allowing the parasite to access the epithelial surface, promoting the growth of pathobiont or pro-inflammatory bacteria, and ultimately contributing to dysbiosis [159].

## Limitations

This review is intended as a narrative synthesis and, as such, reflects literature selection based on author expertise rather than a predefined systematic methodology, introducing potential selection bias. While efforts were made to capture representative and influential studies, some relevant publications may have been omitted. In addition, the rapid pace of discovery in microbiome and parasitology research means that conclusions will require ongoing updating as new data emerge. The geographic representation within the available literature remains uneven due to relatively few studies conducted in endemic settings. Moreover, the methodological heterogeneity across included studies limits quantitative comparison and hinders formal meta-analysis. Variability in parasite detection methods, microbiota profiling approaches, sequencing depth, and analytical pipelines complicates cross-study interpretation.

Lastly, the mechanistic evidence discussed in this review is mainly derived from animal or in vitro models, which may not fully represent human host-parasite-microbiota interactions and therefore requires cautious interpretation. Several fundamental mechanistic questions also remain poorly explored. These include whether (1) helminth-protozoa coinfections influence parasite establishment through direct interference or indirectly via modification of shared microbial communities, (2) which microbiota-derived metabolites or compounds directly affect parasite behavior, development, or reproduction; (3) how parasite antigens or metabolites persist after clearance and continue to modulate host immunity and microbiota and how (4) host genetic factors quantitatively shape the parasite-microbiota-immune interface. Addressing these questions will require innovative, integrated study designs that combine controlled experimental models with well-phenotyped human cohorts.

## Future directions and conclusions

Intestinal parasites exert substantial and heterogeneous effects on the gut microbiota, with helminths and protozoa producing contrasting outcomes, from increased microbial diversity to pronounced dysbiosis [89, 160]. However, current knowledge remains fragmented and disproportionately focused on a limited number of well-studied parasites, overlooking the common occurrence of polyparasitism and clinically neglected parasites that remain underrepresented in endemic regions [26, 65, 102–106]. Furthermore, a major limitation in current parasite-microbiota research is the methodological heterogeneity that undermines reproducibility and cross-study comparability. Discrepancies in study design, ranging from host and parasite species to sequencing platforms, statistical analysis pipelines, and sample sizes, have led to fragmented and often conflicting conclusions [18, 20, 21]. Furthermore, the lack of consistency in microbiota profiling methods and parasite detection techniques introduces confounding variability.

Future research would benefit from adopting more standardised methodological practices. Priority areas include increased consistency in DNA extraction and sequencing protocols, adoption of minimum sequencing depth thresholds sufficient to capture community diversity (e.g., ≥ 10,000 reads per sample for 16 S rRNA profiling), and reporting a core set of diversity metrics like observed richness, Shannon index, and Simpson index, which would improve interpretability across studies. Standardised classification of infection burden using established thresholds, including WHO-based criteria, and systematic reporting of essential covariates, such as age, sex distribution, recent antibiotic exposure, and key dietary characteristics, are equally important. Adopting standardised methodologies that align with those developed by the Human Microbiome Project would improve data transparency and reproducibility [161].

Integrative multi-omics approaches, such as metagenomics, metabolomics, and immune profiling, represent an important avenue for advancing mechanistic understanding of parasite-microbiota-host interactions [113, 162]. However, their implementation remains limited, particularly in parasite-endemic settings where financial and infrastructural constraints limit feasibility. In these contexts, phased and targeted strategies may be more feasible, with initial emphasis on 16 S rRNA profiling, targeted metabolomic analysis of SCFAs and related metabolites, and immune phenotyping using established flow cytometric methods, which provides a balance between depth and accessibility.

Progress in these areas is achievable through shared expertise, infrastructure, and analytical capacity, which helps bridge these gaps in these resource-limited endemic settings. Additionally, emerging analytical and experimental approaches may further advance the field. Machine learning frameworks hold promise for identifying microbiota signatures associated with susceptibility to infection or response to treatment [163]. Using spatial metagenomics and single-cell approaches may help clarify the physical and functional relationships between parasites, epithelial-associated microbiota, and host immune cells.

Beyond these methodological improvements, a comprehensive approach must also consider the broader ecological and host-specific context. Investigating the stark differences between urban and rural populations can serve as a natural experiment to disentangle the effects of factors like sanitation, lifestyle, and antibiotic exposure. Critically, the role of host genetics in determining individual outcomes remains largely unexplored [164]. Future studies incorporating data on polymorphisms in immune pathways (e.g., NOD2, TLRs, IL10), FUT2 secretor status, and HLA types are needed to explain the substantial heterogeneity in microbiota responses to parasitic infection observed across populations [165]. Moreover, systematically exploring the role of diet as a key modulator of nutrient availability for both parasites and microbes is crucial. By integrating data on host genetics, geography, diet, and multi-species infections, we can build a more comprehensive model of this crucial relationship. Longitudinal study designs would be valuable for capturing microbiota development, infection dynamics, and long-term health consequences.

Finally, while clinical translation remains speculative given the current evidence base, parasite-microbiota research raises important translational questions that warrant future investigation. These include whether baseline microbiota composition influences susceptibility to parasitic infection, whether microbiota-targeted interventions could accelerate recovery following antiparasitic treatment, and whether persistent symptoms after parasite clearance may be linked to unresolved microbiota alterations. Addressing these questions will require carefully designed context-appropriate intervention studies.

## Supplementary Information

Supplementary Material 1.

## Data Availability

No datasets were generated or analysed during the current study.

## Abbreviations

ADI: Arginine deiminase
EPG: Number of parasites per gram
ESPs: Excretory-secretory products
GPI: Glycosylphosphatidylinositol
Ig: Immunoglobulins
IL: Interleukin
IPI: Intestinal parasitic infections
NO: Nitric oxide
SCFA: Short-chain fatty acids
STH: Soil-transmitted helminths
TGF-β: growth factor-β
Th2: Type 2 immune response
TLR: Toll-like receptors
TNF: Tumour necrosis factor
Treg: Regulatory T cells
