# Supplementary material for: The dual role of intestinal parasites in shaping human gut microbiota: distinguishing helminthic and protozoan dynamics
Source: Gut Pathog. 2026 Mar 2;18:28. doi: 10.1186/s13099-026-00815-7 (PMC13059267; doi:10.1186/s13099-026-00815-7)
Supplement: Supplementary file 1 — Supplementary Material 1. [file 13099_2026_815_MOESM1_ESM.pdf]

Supplementary Table S1: Summary of the influence of helminth, protozoa and their coinfections on the gut microbiota of human.

Parasite Species	Country	Study Design	Population	Sample Size	Covariates	Methods	Key Findings	Key Taxa Shifted	References
**Helminths**									
A. lumbricoides	Mexico	CS	Adult & children	63	Age and gemder were not considered. Not taken antibiotics and anthelmintic within 2 years.	16S rRNA gene (V4) amplicon sequencing	Gut microbiota co-occurance network differed substantially between parasitised and non-parasitised individuals. Adults showed ~48% differences, whereas children showed ~18% smaller change.	**Parasitised:** ↓ *Coprococcus, Dialister*	[32]
	Thailand	CS	Children (2-18 years) and young adults (19-24 years);	30	Infection intensity was recorded. Age, gender and diet was not used as covariates in analysis. Antibiotic usage were not stated.	16S rRNA gene (V4) amplicon sequencing	Human host microbiota did not differ significantly with infection intensity.	**Ascaris-infected:** ↑ Firmicutes, Bacteroidetes, Proteobacteria**;** ↓ Ruminococcaceae m Lachnospiraceae **Uninfected:** ↑ *Streptococcus*	[54]

T. trichiura	Malaysia	CS & LG	Indigenous (Orang asli) community	67	Urban/rural status served as lifestyle covariates. Blood and nutritional variable, age, gender, overall helminth status, pre-deworming *T. trichiura* egg count were assesed as covariates. No recent history of anthelminthic and antibiotic use within 6 months. No fever or diagnosed with chronic diseases. 5 samples were infected with *E. histolytica* and *G. duodenali*s.	16S rRNA gene (V4) amplicon sequencing; whole blood RNA-Seq	*T. trichiura* infection shows significant variation in microbial beta-diversity relative to diet or blood profiles compared to uninfected urban controls. The *T. trichiura* burden have stronger effect on whole-blood profile compared to diet. While diet differed (higher calories and fiber intake in urban group), *T. trichiura* infection explained more gut microbiota variance.	Following albendazole deworming, significant changes in serum zinc/iron associated with changes in various abundance of microbial taxa independent of dietary metal intake.	[48]

	Tanzania	CS	Women (23-45 years)	10	The study controlled for broad baseline criteria (similar diet, no HIV, no diarrhea, no fever, no antibiotics or anthelmintic treatment in the prior 3 months)	Shotgun metagenomic sequencing	No significant difference in microbial alpha diversity was observed between infected and non-infected women. Significant separation in overall microbial community structure was found between infected and non-infected groups	**Infected:**↑ Firmicutes/Bacteroidetes ratio; ↓*Weisella cibaria, Leuconostoc citreum, Leuconostoc lactis* **Non-infected:** ↑ *Prevotella, Ruminococcus*	[49]
**Hookworms**	Australia	LG	Adult with celiac disease on a long-term gluten-free diet	8	Age and gender are not stated. Volunteers were under gluten-free diet.	16S rRNA (V1-V3 & V3-V5) gene pyrosequencing	A minor non-significant increase in microbial species richness between at T8 compared to T0. No significant change in Shannon diversity. No detectable changes in overall community structure.	No significant shifts.	[47]
	Australia	LG	Individuals with coeliac disease	6	Biopsy samples were collected instead of faecal.	16S rRNA gene (V3-V4) amplicon sequencing	Significantly higher microbial richness prior to gluten exposure (T0) compared to controls; Increased diversity and evenness post-hookworm infection and gluten microchallenge (T24) compared to the T0 group.	**T0-controls:** ↑ Actinobacteria, Actinomycetales, Lactobacillales; **T0-T24:** ↑ Bacteroidia**,** Flavobacteriia**,** Bacteroidales**,** Flavobacteriales	[166]

The Netherlands	RCT	Adult aged 18-45 years (8 males and 12 female)	20	No antibiotic use 6 weeks prior.	16S rRNA gene sequencing	No changes in bacterial diversity in acute infection (weeks 0-8); Microbial Chao1 species richness increased significantly during the established infection phase (weeks 8-20).	No associations between larval dosage; ↑ *Barnesiella* in individuals with severe gastrointestinal symptoms.	[51]
United Kingdom	RCT	Adult with relapsing multiple sclerosis (RMS) between 18-64 years (51 females and 22 males)	73	RMS adults were clinically stable prior 12 months and not subjected to immune-modulatory treatment. Age and gender were considered in analysis.	16S rRNA gene (V3-V4) amplicon sequencing	Higher alpha diversity and a significant increase in beta diversity in the infected group compared to the individual receiving placebo treatment.	↑ *Tenericutes* (RF39, Izimaplasmatales) ; ↓ *Roseburia*, *Dorea*, *Tyzzerella*, *Anaerostipes*, *Agathobacter*	[167]
Mozambique	CS	Individuals aged 5-88 years	89	Infection intensity was recorded. Age and gender was considered in certain analysis. Diet and antibiotics were not reported as covariates.	16S rRNA gene (V3-V4) amplicon sequencing	No significant differences.	No significant shifts.	[52]
**Pathogenic protozoa**								

G. duodenalis	Côte d'Ivoire	CS	Individuals between age 1-74 years	20	Majority were female (n=14) compared to male (n=6). A subsets of them have clinical symptoms (n=7) and febrile (n=1). Antibiotic use was not available.	Temporal Temperature Gradient Gel Electrophoresis (TTGE); qPCR	Significant differences in overall bacterial community structure.	↑ *Escherichia coli*	[108]
	Brazil	CS	Preschool Children between 2-6 years	30	Dietary intake or antibiotic use were not reported.	High Efficiency Microarray; Quantitative PCR (qPCR)	Significantly higher bacterial species diversity.	No significant shifts.	[57]
	Iran	CS	Individuals between 5-73 years	17	Majority of the participants were male (n=13) than female (n=4). Data on clinical symptoms and parasite assemblage were available. Diet or antibiotic	Shotgun metagenomic sequencing	No significant differences in alpha diversity between healthy, asymptomatic and symptomatic groups; Significant microbial compositional shift in beta diversity between controls and overall infected groups, but not across all three groups.	**Infected-control:** ↑ Actinobacteria, Proteobacteria, *Enterococcus faecium;* ↓ Bacteroidetes**,** *Prevotella mizrahii***; Symptomatic:** ↑ *Parolsenella* ***catena,*** *Mitsuokella jalaludinii***;**	[56]

					usage were not stated.			**Asymptomatic:** ↑ *Alistipes shahii*, *Gordonibacter pamelaeae*	
E. histolytica	New Delhi	CS	Patients diagnosed with dysentery	17	Patients age range between 21-40 years. Patients with recent antibiotic or anti-amoebic drug use and recent gastrointestinal symptoms was excluded. Detail on diet was not included.	qPCR	n.s.	↑ *Bifidobacterium*; ↓ Bacteroides, Clostridium coccoides, Clostridium leptum, Lactobacillus, Campylobacter, Eubacterium	[61]
	Cameroon	CS	Adult (26-78 years)	64	Age, gender, subsistence mode and location were considered. Diet survey was recorded.	16S rRNA gene (V5-V6) amplicon sequencing	Significantly higher alpha diversity and lower beta diversity.	↓ *Prevotella copri*, *Prevotella stercorea*; ↑ Clostridiales, Ruminococcacae	[58]

					No antibiotic use was stated.				
	Japan	CS	Patients with amoebic colitis or extraintestinal manifestations and asymptomatic	77	Clinical variables such as site of infection, presence of trophozoites or cysts, symptomatic or asymptomatic cases were recorded. Detail on dietary intake and antibiotic use were not available.	16S rRNA gene (V3-V4) amplicon sequencing	No significant differences in overall alpha diversity between asymptomatic and symptomatc groups. Significantly diverse and differ in microbial community in symptomatic groups.	↑ Ruminococcaceae, Coriobacteriaceae, Clostridiaceae; ↓ Streptococcaceae	[60]
Cryptosporidium spp.	Ukraine	CSR	Case study of single paediatric patient with comorbidities	1	Severe immunodeficiency and comorbid with hepatic disease. Patients had multiple rounds of antiparasitic therapies.	16S rRNA gene sequencing	Significant reduction in microbial diversity and richness, and microbial compositional shift toward pro-inflammatory taxa.	↑ Pro-inflammatory bacteria (e.g., Enterobacteriaceae); ↓ Commensal & beneficial bacteria (e.g., Bifidobacterium, Lactobacillus)	[63]

	Italy	CSR	Case study with underlying pathologies	5	Four patients were immunocompromised, and 1 was immunocompetent. Some were receiving antibiotics at the time of sampling.	16S rRNA gene (V3-V4) amplicon sequencing	Moderate to severe cryptosporidiosis reduced the alpha diversity compared to healthy controls. Mild infections showed similar alpha diversity to controls.	**Moderate/Severe:** ↑ *Enterococcus*; ↓ *Bifidobacterium*, *Gemmiger*, *Blautia*; **Mild:** ↑ *Agathobacter*, *Sutterella;* ↓ *Gemmiger*	[64]
**Coinfections**									
T. trichiura or coinfections (*T. trichiura* and *A. lumbricoides*)	Ecuador	CS & LG	Children (8-14 years)	97	Participants were recruited from 3 rural villages. Not taken antibiotics or anthelmintics 3 months prior, afebrile and asymptomatic.	16S rRNA gene (V3-V5) amplicon sequencing	No significant shift in *T. trichiura* infection compared to uninfected children. Reduced bacterial diversity in coinfected children compared to the other groups. Althelmintic treatment of *T. trichiura* did not alter microbial composition.	**Coinfected=** ↓ *Clostridia sensu stricto*; ↑ *Streptococcus* spp. (in some individuals)	[29]
T. trichiura coinfected with *A. lumbricoides* or hookworm or both	Malaysia	CS	Indigenous (Orang asli) community of varying age	51	Participants were recruited from 2 village with a median age of 60.5. No antibiotic history stated.	16S rRNA gene (V4) amplicon sequencing	Higher bacterial richness and significant differences in microbial composition.	↑ Paraprevotellaceae, Mollicutes, Bacteroidales, Alphaproteobacteria; ↓ Bifidobacterium	[22]

Soil-transmitted nematode (*T. trichiura*, *A. lumbricoides* or hookworm	Sri Lanka	CS	Individuals of varying age	76	Participants were recruited from 9 villages and had not received antibiotic treatment 6 months prior.	16S rRNA gene (V3-V4) amplicon sequencing	No significant differences in richness and Shannon diversity between groups. Significant increase in community composition in helminth-infected and treated groups compared to uninfected.	**Helminth-infected:**↑ Verrucomicrobiaceae, Enterobacteriaceae, *Lactococcus*, *Akkermansia*; **Treated:** ↑ Clostridiacea**,** **Untreated:** ↑ Leuconostocacea	[33]
STH (*T. trichiura*, *A. lumbricoides* or hookworm	Indonesia	RCT	Adult and children	150	Age and gender were considered.	16S rRNA gene (V1-V3) pyrosequencing	No significant changes in Shannon diversity and microbial composition with helminth infection. Significant different in composition between albendazole and placebo in participants who remained infected after treatment.	**Post-albendazole:** ↑ Actinobacteria; ↓ Bacteroidetes	[34]
STH (*A. lumbricoides*, *T. trichiura and* hookworms)	Indonesia & Liberia	CS & LG	Individuals aged 6-59 years	402	Age range between 6 and 59 years. More female (n=53) than male (n=45).	16S rRNA gene (V1-V3) amplicon sequencing; Shotgun metagenomic sequencing for functional analysis	**Liberia:** Increased alpha diversity and significant differences in beta diversity in STH-infected individuals compared to uninfected controls. **Indonesia:** Microbial richness was slightly higher in infected individuals but no significant differences in beta diversity in infected individuals. **Deworming/self-clearing:**	**Overall helminth-infected:** ↑ *Olsenella*, *Flavonifractor*, *Enterococcus*, *Allobaculum*; ↓ Lachnospiracae incertae sedis **Deworming:** Taxa like *Enterococcus* increased over	[25]

							Microbiome did not fully revert toward the uninfected community state	time independently of infection clearance	
STH (*A. lumbricoides, N. americanus* and both)	Western Kenya	CS & LG	Individuals aged 3-84 years	43	Participants were recruited from 5 rural villages. Age-matched controls were used for pretreatment comparisons. The details on the diet were not stated. No control on antibiotic and antiparasitic use. No worm burden data measured.	16S rRNA gene sequencing	No significant differences in overall diversity pre-treatment. Significant differences were observed post-treatment and clearance.	**Post-treatment:** ↑ Clostridiales; ↓ Enterobacteriales	[36]

A. lumbricoides coinfected with either *T. trichiura* or *Enterobius vermicularis;* coinfected with *Giardia*	Colombia	CS	Children	23	Diet was implied to be similar. No details on antibiotic use.	16S rRNA gene (V4) amplicon sequencing	***Giardia*-only:** Reduced Shannon and species richness but were not statistically significant. ***Cryptosporidium*-only:** Reduced Shannon diversity, but not statistically significant. ***Giardia* + Helminth:** Reduced Shannon's diversity and increased in observed bacterial species. But the results were not statistically significant.	***Giardia*-only:** Shifted to enriched in *Prevotella* spp. (Enterotype II); ↑ *Ruminococcus, Prevotella* ***Cryptosporidium*-only:** ↑ *Bacteroides* **Giardia + Helminth:** Shifted to enriched in *Prevotella* spp. (Enterotype II); ↑ *Prevotella*, unclassifed Porphyromonadaceae, *Alloprevotella*	[30]
Protozoa*: B. hominis, Entamoeba coli, Entamoeba dispar, Endolimax nana, Iodamoeba butschlii, Chilomastix mesnili, G. duodenalis;* Helminths: *Hymenolepis*	Mexico	CS	Mother-child pairs	92	Children age range from 2 to 20 months and breastfed for 6-12 months. Twelve delivered by C-section and 34 by vagina. Mothers age range from 18 to 47 years. No	16S rRNA gene (V4) amplicon sequencing	No significant differences in alpha diversity between parasite-exposed and nonexposed groups. Parasitic exposure shows significant variation in bacterial structure across age groups. Parasite-negative children (exposed to parasite-positive mothers) under 1 year of age show significantly	**Unweaned infants (<5 months):** ↑ *Bifidobacterium, Bacteroides*; **Nonexposed infants:** ↓ Pseudoramibacter, Eubacterium, Prevotella, Oscillospira; **Weaned infants**	[168]

nana, A. lumbricoides.					history of antibiotic or drug use, gastrointestinal or inflammatory symptoms in 6 months prior to sampling.		dissimilar in bacterial community.	**(>1 years):** ↑ , Bacteroidia, Actinobacteria, *Clostridia*, Coriobacteriia	
Protozoa: *E. histolytica/dispar, G. duodenalis, Entamoeba coli, Endolimax nana;* Helminths: Hookworms (*Ancylostoma duodenale*) and *Hymenolepis nana*	West Africa	CS	Children	1,204	Confounding variables were addressed. Children with a history of antibiotics for 3 months prior were excluded. Subsequent analyses were adjusted for cofounding variables such as age, history of vitamin A intake and factors associated with risk of intestinal parasites.	16S rRNA gene (V3-V5) amplicon sequencing	Alpha diversity was largely unaffected, but significant differences in overall community composition in protozoan infections; multi-species infection induced more pronounced shifts in microbial composition.	↑ *Prevotella*, *Campylobacter*, 2 *Clostridium* clades; ↓ Collinsella, Lactobacillus, Ruminococcus, Veillonella, Clostridium clade	[169]

A. lumbricoides, T. trichiura and *hookworm*	Malaysia	CS & LG	Indigenous (Orang asli) community	351	Participants were recruited from 5 OA villages.	Shotgun metagenomic sequencing	Higher species richness in infected subjects. The OA village has the largest effect size on the microbial diversity and composition, and high-burden helminths exhibited greater microbial diversity.	**Orang Asli-urban:** ↑ *Agathobaculum, Ruminococcus_E*; ↓ *Bacteroides*	[90]
A. lumbricoides* and *Trichuris trichiura	Malaysia	CS	Children (3-11 years); Male=4, Female=4	8	Participants recruited from 2 rural villages. Asymptomatic and no recent antihelmintic/antibiotic use. Age and gender were assessed.	16S rRNA gene (V3-V4) amplicon sequencing	Higher bacterial abundance and diversity in STH-positive individuals compared to the STH-negative groupbut were not significant	**STH-positive:** ↑ Bacteroidetes, Spirochaetes, *Faecalibacterium, Succinivibrio* **STH-negative:** ↑ Firmicutes, Proteobacteria, Actinobacteria, Clostridium, Bifidobacterium	[170]
STH (*A. lumbricoides, T. trichiura*, hookworms) and *Schistosoma mansoni*	Ethiopia	CS & LG	Children	138	Children aged above 5 years. Sixty-nine children had a history of deworming in the past 6 months.	16S rRNA gene (V4) amplicon sequencing	No significant difference in alpha diversity and microbiata composition between STH-infected and non-infected children. Children with *T. trichiura* had significantly lower microbial diversity and differences in microbial composition. *T. trichiura* egg count significantly asscociated with taxa, *Agathobacter* compared to age, weight and heigth.	***Trichuris*-infected:** ↑ Agathobacter, Lachnospiraceae; **Deworming** had significantly higher Firmicutes	[171]

Giardia duodenalis and STH (*A. lumbricoides*, Hookworms, *T. trichiura, S. stercoralis*)	Argentina	CS	Children (3-8 years)	37	Asymptomatic children. Giardia burden was considered. Age, diet, and gender were not considered as primary covariates. All children had not received antiparasitic or antibiotic treatment.	Shotgun whole-genome sequencing	Significantly lower Shannon diversity in Giardia-only group with correlation with Giardia burden when compared with uninfected children. Increase diversity in helminth-only group compared to uninfected chldren, while Giardia + helminth coinfections exhibited decreased diversity relative to helminth-only.	**Giardia-only:** ↑ *Bacteroidales*, *Prevotella*<br>**Heliminth-only:** ↓ Actinobacteria<br>**Giardia + helminth:** ↑ *Prevotella*	[27]
STH (*A. lumbricoides*, *N. americanus*, *T. trichiura* and *S. stercoralis*)	Cameroon	CS	Adult and children	575	Subsistence strategy, parasite burden, blood parasite,immune marker. Age, gender and region were considered.	16S rRNA gene (V4) amplicon sequencing for faecal bacterial communities; Shotgun metagenomic sequencing for higher taxa resolution and functional profile	Significant positive correlation with alpha diversity with helminth burden. Microbial composition was differed in helminth infections compared to uninfected.	**Helminth-positive:** ↑ *Prevotella*, *Succinivibrio, Treponema*, Bacteroidales, CF231, Anaerovibrio<br>**Helminth-negative:** ↑ *Ruminococus bromii*	[28]

T. trichiura or coinfections (*T. trichiura* and *A. lumbricoides*)	Tanzania	CS	Mother-child pairs;<br>Mother (23-45 years) and children (1.5-2.6 years)	56	Participants were screened to have similar diets and to be free of recent antibiotics, anthelmintics, HIV, fever, diarrhea, malaria, diabetes or pregnancy.	16S rRNA gene (V3-V4) amplicon sequencing	In mothers, helminth-positive individuals had significantly higher alpha diversity than uninfected controls.<br>Faith's diversity was significantly higher in infected than uninfected, while observed OTUs and Shannon index showed non-significant trends toward higher diversity. Beta diversity demonstrated infection-associated clustering in both groups, with significant differences in community structure.	**Mother-children:**↓ SCFAs producers, carbohydrate-degrading bacteria;<br>**Mother:** ↑ *Campylobacter***,** *Methanobrevibacter***;**<br>**Children:** ↑ *Enteroccocus*	[45]

Any protozoa (*Blastocystis sp.* (various subtypes), *D. fragilis, G. duodenalis, C. parvum*)	Italy	CS	Diarrheal patients	57	Blastocystis subtypes (ST1-ST4), age class, gender, geographic origin, eosinophilia levels, SARS-CoV-positivity. Data on diet and antibiotic use were unavailable.	16S rRNA gene sequencing	Did not report diversity but the relative abundance of bacterial species.	<u>Most Prevalence Taxa</u> **Blastocystis-only:** Escherichia fergusonii ATCC 35469 **Dientamoeba fragilis:** E. fergusonii **G. duodenalis:** Enterococcus faecium **C. parvum:** E. fergusonii **Blastocystis + D. fragilis:** E. fergusonii **Blastocystis + C. parvum:** E. fergusonii **Blastocystis + D. fragilis + G. duodenalis**:Eubacterium rectale ATCC 33656 **Blastocystis + G. duodenalis:** Phocaeicola dorei	[110]

Any helminths (*A. lumbricoides, T. trichiura, Hymenolepis diminuta*) or any protozoa *(E. histolytica, Dientamoeba fragilis, G. duodenalis, C. parvum*)	Indonesia	CS	Children (Age: 7-12 years.	140	Socioeconomic status. Age, gender, anthropometry, no recent antibiotic use, household sanitation and hygiene. intestinal parasite infection status were adjusted.	16S rRNA gene (V3-V4) amplicon sequencing	Parasite infection was more prevalent in low-SES children<br>Higher bacterial richness and diversity in low-SES children<br>High-SES children had lower diversity and the microbiomes consistent with more 'Western-like'<br>Clear microbial community separation between the two SES groups<br>Bacterial richness and diversity were similar between helminth or protozoa infections<br>SES explained a significant proportion of microbiome variation after accounting for age and parasite infection	↑ **Low-SES children:** *Prevotella, Succinivibrio, Megasphaera, Faecalibacterium*<br>↑ **High-SES children:** *Bacteroides, Bifidobacterium, Blautia, Ruminococcus*	[38]

n.s.: not stated; CS: cross-sectional study; LG: longitudinal study; RCT: randomized-controlled trial; CSR: case study; SES: socio-economic level
